# Emotion meets coordination: Designing multi-agent LLMs for fine-grained user sentiment detection on social media

**DOI:** 10.1371/journal.pone.0342053

**Published:** 2026-02-09

**Authors:** Hao Dong, Zuowen Bao, Muze Li, Zhengfeng Yang

**Affiliations:** 1 Department of Digital Media Art, Jiangsu Second Normal University, Nanjing, Jiangsu, China; 2 Department of Design, Sungkyunkwan University, Seoul, South Korea; 3 Department of Visual Design, Hanyang University, Ansan, South Korea; University of Sargodha, PAKISTAN

## Abstract

Social media platforms have become central channels for emotional communication, posing new challenges for fine-grained sentiment analysis due to their high contextual variability, multimodal content, and pervasive ambiguity. Traditional end-to-end sentiment models often struggle to capture compositional or conflicting emotional cues in user-generated texts. This study presents a modular multi-agent architecture for sentiment analysis, implemented with the LLaMA-3.3-70B-Instruct model and guided by system-level design principles. The framework decomposes emotion inference into three coordinated stages, perception, reasoning, and resolution, each managed by a specialized agent trained with parameter-efficient tuning strategies. A meta-agent mediates conflicting predictions through a coordination protocol based on confidence estimation and discourse consistency, enabling adaptive consensus formation. Evaluations on the GoEmotions v2, SemEval-2024, and Twitter benchmarks demonstrate that the proposed system achieves higher accuracy, robustness, and interpretability compared with existing baselines. These findings indicate that architectural decomposition combined with collaborative reasoning enhances reliability and transparency in sentiment analysis, offering a scalable pathway toward intelligent and emotionally aware computational systems.

## Introduction

The growing prevalence of social media platforms as key channels for personal expression, political discourse, and public sentiment has created a pressing need for computational methods that can accurately interpret emotional signals embedded in user-generated content. Unlike formal texts, social media posts are often terse, unstructured, and multimodal, exhibiting informal vocabulary, emojis, code-switching, abbreviations, and slang [1]. The emotional landscape of these texts is correspondingly complex, and is rarely limited to simple positive or negative polarity. Users frequently express multiple emotions in a single post, blend affective tones with sarcasm, or use ironic statements to imply sentiment contrary to their literal wording. Consequently, fine-grained sentiment detection, where systems are required to identify nuanced emotions such as joy, fear, disgust, anticipation, or confusion—has become an essential subtask of affective computing in social media contexts [2].

However, this task poses considerable challenges. The surface lexical cues of emotion are often misleading, and syntactic indicators such as negation or conditionality may alter the intended emotional interpretation. Moreover, sentiment cannot be decoupled from context; identical phrases may convey different emotions depending on preceding utterances, user background, or cultural expectations. For instance, the phrase “Thanks a lot” may be genuine or sarcastic depending on tone, capitalization, or context. Current sentiment analysis models often lack the capacity to reason about such contextual subtleties, leading to systematic misclassification [3], particularly in emotionally ambiguous or code-mixed utterances. Furthermore, while many applications demand real-time or scalable analysis, achieving both computational efficiency and emotional fidelity remains a difficult trade-off.

Despite notable advances brought by large language models (LLMs), particularly in instruction-following and few-shot learning, existing sentiment analysis systems typically rely on single-model paradigms that collapse the entire reasoning process into a single forward pass. This architecture inherently limits modular interpretability and flexibility, especially when dealing with complex emotional constructs or competing affective cues [4]. Moreover, models such as RoBERTa, DeBERTa, or ChatGPT often exhibit an over-reliance on surface tokens and underperform on tasks involving sarcasm, irony, or mixed emotions. In scenarios where multiple emotions co-occur or conflict, the lack of an explicit resolution mechanism leads to degraded performance and reduces the explanatory accountability of model outputs. These limitations suggest that a monolithic approach, however powerful, may not suffice for fine-grained emotion detection in highly variable real-world settings [5].

In this work, we propose a novel multi-agent architecture for sentiment analysis that builds upon the generative reasoning and representational strengths of the LLaMA-3.3-70B-Instruct model. Rather than treating sentiment detection as a static classification task, our framework distributes responsibility across specialized agents [6], each designed to handle a distinct aspect of emotional reasoning. This includes agents for perceptual cue extraction (e.g., emotion words, emojis, stylistic markers), contextual interpretation (e.g., sarcasm detection, implicit sentiment inference), and resolution of conflicting predictions [7]. These agents are instantiated through targeted LoRA-based fine-tuning of LLaMA submodules, allowing each agent to develop localized expertise while benefiting from shared foundational knowledge. This structure enables dynamic collaboration among agents, thereby better capturing the heterogeneous nature of affective expressions across social media posts.

The architecture is operationalized as a three-stage pipeline encompassing perception, reasoning, and coordination. The perception stage identifies and annotates affective elements and candidate sentiment spans. The reasoning stage contextualizes these elements within the broader discourse, adjudicating ambiguities and assessing compositional sentiment. Finally, the coordination stage resolves disagreements between agents by leveraging a meta-agent that calibrates outputs based on confidence scores, historical context, and potential contradictions. This collaborative protocol is designed to simulate human-like affective reasoning, wherein different cognitive modules interact to produce a coherent emotional judgment. Importantly, the modularity of the system supports both transparency and extensibility, making it adaptable to new domains, emotion taxonomies, or multilingual settings [8].

To evaluate the efficacy of our approach, we conduct experiments on a suite of high-quality benchmarks, including GoEmotions v2, SemEval-2024 Task 10, and Twitter datasets annotated with fine-grained emotion labels. These datasets represent a diverse range of emotional expressions, communication styles, and platform-specific phenomena such as irony or troll behavior. Our results show that the multi-agent architecture consistently outperforms strong baselines, including both transformer-based models and API-level LLMs like GPT-4 Turbo. Moreover, we perform detailed ablation studies to quantify the individual contributions of each agent and analyze failure modes associated with agent disagreement, ambiguous labels, and cross-cultural variation. The system demonstrates improved robustness under adversarial perturbations and higher inter-annotator agreement in human evaluation tasks [9].

Beyond empirical gains, the proposed framework contributes to the broader research agenda of integrating symbolic reasoning structures into neural architectures. By treating emotion understanding as a coordinated task involving competing hypotheses and conflict resolution, our method offers a template for future NLP systems that prioritize interpretability and modular reasoning. This aligns with recent trends in multi-agent dialogue modeling, deliberative planning, and decentralized inference, suggesting a broader applicability of our approach to tasks such as hate speech detection, mental health analysis, and computational social science [10]. In particular, the inclusion of an explicit disagreement-resolution agent opens new opportunities for examining affective conflict and emotional polyphony in digital discourse.

In summary, this paper makes several key contributions. First, we present the first large-scale multi-agent sentiment analysis system grounded in an LLaMA-3.3 backbone, tailored to address the complexities of social media emotion detection. Second, we introduce a novel coordination protocol that resolves prediction conflicts among agents, increasing system reliability and enabling finer granularity in sentiment output [11]. Third, we empirically demonstrate state-of-the-art performance across multiple datasets and metrics, including classification accuracy, macro-F1, robustness scores, and agent agreement rates. Lastly, we provide extensive qualitative and quantitative analyses to support the interpretability, scalability, and cross-linguistic adaptability of our system. Taken together, these contributions represent a significant advance in the landscape of sentiment analysis research.

Through this work, we aim to shift the focus from monolithic sentiment classification pipelines to cooperative architectures that reflect the multifaceted nature of human emotion. We believe that such a reorientation is necessary not only for improving performance but also for enhancing the interpretability and societal accountability of emotion-aware systems. As social media continues to evolve as a global medium of emotional expression, systems capable of nuanced, context-aware, and robust sentiment detection will be essential to applications ranging from customer service automation to policy monitoring and mental health support [12]. We envision this research as a foundation for future developments in affective multi-agent NLP systems that are both technically rigorous and ethically attuned to the complexities of human communication.

## Related work

### Background: Polarity-based sentiment analysis (positive/neutral/negative)

Early sentiment analysis research primarily focused on polarity classification, categorizing text as positive, neutral, or negative. This framework proved effective for tasks such as opinion mining and product review analysis [13], but it fails to capture subtle emotional gradients, irony, or mixed expressions common in social media discourse. Moreover, polarity-based models overlook compositional linguistic cues (e.g., contrastive conjunctions or sarcasm) and cultural affective nuances, thereby motivating a transition toward richer emotion taxonomies and hierarchical sentiment representations.

### Fine-grained sentiment analysis

Fine-grained sentiment analysis extends traditional polarity classification by identifying specific emotions such as joy, sadness, anger, fear, surprise, and disgust. This paradigm shift from coarse polarity to affective granularity has been driven by emerging applications in mental health monitoring, hate speech detection, and social media analytics, where systems must detect subtle affective signals rather than general sentiment polarity [13].

In response, diverse emotion taxonomies and frameworks have been developed. The Ekman model, Plutchik’s wheel, and the Geneva Emotion Wheel establish psychologically grounded emotion structures, while data-driven resources such as GoEmotions define 28 emotion categories derived from Reddit posts [14]. These taxonomies underpin supervised multi-label classification approaches. However, fine-grained emotion detection remains challenging due to label imbalance, emotional co-occurrence, and contextual ambiguity across cultures and social domains.

Early neural architectures based on RNNs and CNNs have been succeeded by transformer-based models such as BERT or RoBERTa fine-tuned on emotion corpora. Despite achieving higher macro-F1 scores [15], these models often overfit lexical features and struggle with contextual ambiguity, sarcasm, and multi-emotion overlap. Consequently, researchers are exploring modular and multi-step reasoning frameworks that improve interpretability and capture inter-emotion dependencies [16].

Recent research (2024–2025) has further advanced fine-grained emotion understanding through multimodal and cross-domain modeling. Liu et al. [17] proposed MDKAT, a multimodal decoupling framework with knowledge aggregation and transfer for video emotion recognition. Xu et al. [18] introduced diversified prompt composition for image emotion classification, while Guo et al. [19] presented a unified generative framework for multimodal emotion understanding. Beyond vision, Zhao et al. [20] applied EEG-based emotion recognition using autoencoder fusion and MSC-TimesNet modeling. In the realm of cross-modal sentiment analysis, Ma et al. [21] developed a contrastive fusion network with dynamic semantic diffusion. For education-specific emotion analysis, Liu et al. [22] employed graph neural networks with multi-head cross-attention. Addressing data quality challenges, Jiang and Yin [23] tackled occluded expression recognition using adversarial networks and dictionary learning in robot environments. Beyond vision, Zhao et al. [18] applied EEG-based emotion recognition using autoencoder fusion and MSC-TimesNet modeling, and Li et al. [24] employed reinforcement learning to intervene in negative emotion contagion on social networks. Together, these studies highlight a shift toward knowledge-enhanced, interpretable, and cross-modal emotion reasoning—directions that align closely with the multi-agent sentiment coordination framework proposed in this paper.

### Large language models for sentiment tasks

Large-scale pre-trained language models (LLMs) such as GPT-3, ChatGPT, and LLaMA have revolutionized NLP and shown impressive few-shot and zero-shot capabilities in sentiment classification [25]. These models internalize affective patterns from massive corpora and produce contextually coherent sentiment judgments even for novel or low-resource settings. Their long-context reasoning and implicit world knowledge make them attractive candidates for fine-grained emotion recognition.

Nevertheless, LLMs exhibit several limitations when applied to sentiment tasks. They may produce inconsistent predictions across paraphrased inputs, struggle with compositional sentiment (e.g., “I love the food but hate the service.”), and display unstable emotional calibration. Moreover, their monolithic architectures prevent modular decomposition of sentiment reasoning and hinder interpretability. While prompt engineering and parameter-efficient fine-tuning techniques such as LoRA and AdapterFusion [26] partially alleviate these issues, the underlying reasoning remains opaque. These challenges have motivated the development of hybrid and multi-agent architectures that combine LLM expressiveness with explicit modular reasoning and coordination.

### Multi-agent NLP systems

Multi-agent systems (MAS) have emerged as a promising paradigm for complex NLP tasks requiring distributed reasoning, contextual disambiguation, or collective decision-making. Instead of a single monolithic model, MAS architectures delegate subtasks to specialized agents and integrate their outputs through mechanisms such as debate, voting, or confidence-weighted fusion [27]. This approach reflects human cognitive processes, where perception, memory, and reasoning operate in parallel yet cooperatively.

In sentiment analysis, MAS has been applied to multi-perspective tasks such as dialogue understanding, misinformation detection, and hate speech classification, where independent agents can provide complementary or corrective interpretations. These systems improve calibration, enable modular debugging, and enhance robustness via agent diversity.

However, most existing MAS frameworks either employ static transformer heads without communication or rely on rule-based coordination. Few have integrated modern LLMs into dynamic agent collaborations capable of iterative reasoning and belief revision. Our proposed framework addresses this gap by adopting LLaMA-3.3-70B-Instruct as the backbone for three specialized agents—Perception, Reasoning, and Conflict Resolution—each fine-tuned for distinct roles. By combining LLM-level language understanding with explicit inter-agent coordination, this framework advances fine-grained, interpretable sentiment reasoning across heterogeneous social media environments.

## Task formulation

### Problem formulation

The core task addressed in this work is fine-grained sentiment classification over social media posts, which requires identifying one or more emotional categories conveyed within a given unit of user-generated content. Formally, let the input be a post x∈𝒳, where 𝒳 denotes the space of textual (and optionally multimodal) utterances. Each post is associated with a latent set of emotions y⊆ℰ, where $ℰ=\{e_{1},e_{2},\ldots ,e_{K}\}$ represents the predefined emotion taxonomy, and *K* is the number of emotion categories (e.g., 28 for GoEmotions). The model’s objective is to learn a mapping function $f:𝒳\to 2^{ℰ}$ that returns a subset of relevant emotions for any input *x*.

This problem is inherently multi-label, requiring the model to handle overlapping emotional constructs and recognize implicit sentiment signals. The complexity increases with inputs involving sarcasm, negation, indirect affect, or multi-aspect discourse. Let the model predict a probabilistic distribution $\hat{y}=f(x;\theta )\in [0,1{]}^{K}$, where *θ* represents trainable parameters and ${\hat{y}}_{k}$ is the predicted probability of the *k*-th emotion. We employ a binary cross-entropy loss to optimize the model across multiple labels:

$$L_{\text{bce}}=-\sum_{k=1}^{K}\left[y_{k}\log{\hat{y}}_{k}+(1-y_{k})\log(1-{\hat{y}}_{k})\right]$$ (1)

This formulation supports soft probability calibration and is compatible with threshold-based decision rules at inference time. To adapt the system to noisy labels and class imbalance, we further integrate a dynamic focal loss variant:

$$L_{\text{focal}}=-\sum_{k=1}^{K}\alpha_{k}(1-{\hat{y}}_{k}{)}^{\gamma }\;y_{k}\log{\hat{y}}_{k}$$ (2)

where $\alpha_{k}$ is a class-specific weighting factor and *γ* controls the degree of penalization on well-classified examples. Unlike the static focal formulation, the variant introduces adaptive weighting based on both class frequency *f*_*k*_ and training progression *t*. Specifically, the coefficients evolve as $\alpha_{k}=1\;-\;f_{k}$ and γ=1+log(1+t/T), allowing the model to gradually emphasize minority emotions as training advances. This instance-level adaptation ensures proportional gradient updates and stabilizes convergence across heterogeneous emotion distributions.

In cases where the input includes auxiliary information such as image embeddings $v\in {\mathbb{R}}^{d}$ or thread context $c\in {𝒳}^{n}$, the input representation is extended via a concatenated multi-modal encoding: $x^{\prime }=ENC(x,v,c)$, where ENC denotes a fusion encoder built on cross-attention layers. The mapping function is accordingly redefined as $f:𝒳\times {\mathbb{R}}^{d}\times {𝒳}^{n}\to 2^{ℰ}$. The architecture must therefore operate under partial observability and noisy supervision, requiring robust latent reasoning components [28].

The task formulation also permits hierarchical reasoning by predicting both coarse (Ekman-style) and fine-grained (GoEmotions) categories. We define a dependency matrix $G\in \{0,1{\}}^{K\times C}$, where *C* is the number of coarse categories. Let $z_{c}=\sum_{k:G_{kc}=1}{\hat{y}}_{k}$ denote the aggregated score for coarse class *c*. This dual-level representation supports consistency regularization and is optimized jointly via:

$$L_{\text{total}}=\lambda_{1}L_{\text{bce}}+\lambda_{2}L_{\text{coarse}}+\lambda_{3}L_{\text{focal}}$$ (3)

where $\lambda_{1}$, $\lambda_{2}$, and $\lambda_{3}$ are non-negative scalar weights controlling the relative importance of the fine-grained, coarse-level, and focal loss components, respectively. In our experiments, $\lambda_{i}\in [0,1]$ and are tuned such that $\lambda_{1}+\lambda_{2}+\lambda_{3}=1$, with the default setting $\left(\lambda_{1},\lambda_{2},\lambda_{3})=(0.6,0.2,0.2\right)$ unless otherwise specified.

### Hierarchical emotion taxonomy and graph regularization

The selection of an emotion taxonomy is non-trivial in the context of fine-grained sentiment detection, particularly for social media. We adopt a hybrid taxonomy that combines the empirical breadth of GoEmotions with the psychological structure of Ekman’s basic emotions. Let ℰ be the fine-grained emotion set and 𝒞⊂ℰ the set of six supercategories: joy, sadness, anger, fear, disgust, and surprise. We define a bipartite graph 𝒢=(ℰ,𝒞,A), where adjacency matrix $A\in \{0,1{\}}^{K\times 6}$ maps fine emotions to their coarse parent nodes.

This graph structure enables us to model label dependencies through structured inference. For example, if an emotion such as “gratitude” is predicted with high confidence, then the score for “joy” should also be elevated. During training, we enforce consistency by introducing a graph-based regularizer:

$$L_{\text{graph}}=\sum_{(e_{k},c_{j})\in 𝒢}({\hat{y}}_{k}-{\hat{z}}_{j}{)}^{2}$$ (4)

where ${\hat{z}}_{j}=\sum_{k:A_{kj}=1}{\hat{y}}_{k}$ is the aggregated score for supercategory *c*_*j*_. This regularization ensures coherent predictions across hierarchical emotion paths and encourages low-entropy assignments.

To support inter-label reasoning, we construct a semantic similarity matrix $S\in {\mathbb{R}}^{K\times K}$ where $S_{ij}=\cos(\phi (e_{i}),\phi (e_{j}))$, with ϕ(·) representing pretrained emotion embeddings (e.g., BERT or ConceptNet vectors). We define a smoothness constraint to promote semantically aware classification:

$$L_{\text{smooth}}=\sum_{i,j}S_{ij}\;({\hat{y}}_{i}-{\hat{y}}_{j}{)}^{2}$$ (5)

This term encourages similar emotion classes (e.g., “embarrassed” and “ashamed”) to receive proportionally close probabilities, improving generalization on sparse or ambiguous examples.

The inclusion of graph-based and smoothness regularization terms is theoretically grounded in manifold regularization [29], which enforces local smoothness among semantically or hierarchically related emotions (e.g., “joy” ↔ “excitement”). This mechanism mitigates over-segmentation of affective subclusters and promotes label consistency across the emotion hierarchy by minimizing prediction variance along the intrinsic geometry of the emotion manifold. Accordingly, the smoothness constraint complements the Laplacian term by stabilizing hierarchical dependencies between coarse and fine-grained emotion representations, leading to more consistent and interpretable classification outcomes.

The taxonomy also supports multilingual adaptation. Let $\tau :ℰ\to {ℰ}^{\left(l\right)}$ be a language-specific emotion mapping, where l∈{en,zh,es,…}. This allows us to train multilingual agents on aligned taxonomies while retaining universal semantic grounding. We evaluate taxonomy transferability using cross-lingual divergence:

$$D_{\text{lang}}=\sum_{k=1}^{K}KL(y_{k}^{\left(en\right)}\;\|\;y_{k}^{\left(l\right)})$$ (6)

This facilitates both zero-shot generalization and fine-tuned specialization, allowing the proposed system to adapt to the linguistic and cultural nuances of emotion expression.

### Multi-agent sentiment reasoning setup

The proposed method adopts a three-stage multi-agent reasoning protocol: perception, analysis, and coordination. Each agent is instantiated as a lightweight LoRA-tuned version of LLaMA-3.3-70B-Instruct, specialized via modular objectives. Let $𝒜=\{A_{1},A_{2},A_{3}\}$ denote the set of agents, where each *A*_*i*_ computes partial predictions ${\hat{y}}^{\left(i\right)}\in [0,1{]}^{K}$. The global output is aggregated via a coordination function $g:\{{\hat{y}}^{\left(i\right)}{\}}_{i=1}^{3}\to [0,1{]}^{K}$, learned through confidence calibration and meta-inference.

The perception agent *A*_1_ performs span-level extraction. Given an input *x*, it outputs a matrix $P\in {\mathbb{R}}^{n\times K}$, where *P*_*jk*_ represents the attention score of token *j* toward emotion *k*. These scores are normalized via a token-level softmax:

$$\alpha_{jk}=\frac{\exp(P_{jk})}{\sum_{t=1}^{n}\exp(P_{tk})}.$$ (7)

The reasoning agent *A*_2_ processes contextual cues and outputs intermediate logits $r=f_{r}(x;\theta_{r})\in {\mathbb{R}}^{K}$. It employs masked contrastive objectives to disambiguate conflicting emotions:

$$L_{\text{contrast}}=-\log\frac{\exp(sim(r_{k},y_{k}^{+}))}{\sum_{j}\exp(sim(r_{k},y_{j}^{-}))},$$ (8)

where $y_{k}^{+}$ and $y_{j}^{-}$ are positive and negative prototypes derived from emotion centroids, and sim(·) is cosine similarity.

The resolver agent *A*_3_ takes the output of *A*_1_ and *A*_2_, along with disagreement indicators $\delta_{k}=|{\hat{y}}_{k}^{\left(1\right)}-{\hat{y}}_{k}^{\left(2\right)}|$, and generates a final prediction via meta-weighted fusion:

$${\hat{y}}_{k}=\sigma (w_{k1}{\hat{y}}_{k}^{\left(1\right)}+w_{k2}{\hat{y}}_{k}^{\left(2\right)}),\;\text{where }w_{ki}=\frac{1}{1+\delta_{k}}.$$ (9)

This adaptive weighting mechanism ensures that the agent most confident about a given emotion contributes more to the final decision. The weights are further calibrated using temperature-scaled softmax over agreement scores.

To maintain representational diversity during the early phase of training and prevent premature convergence among agents sharing the same initialization, we introduce a mild diversity-preserving regularizer:

$$L_{\text{div}}=\sum_{i\neq j}{\left\|{\hat{y}}^{\left(i\right)}-{\hat{y}}^{\left(j\right)}\right\|}_{1}$$ (10)

Contrary to a purely “disagreement-promoting” objective, this regularizer does not enforce arbitrary divergence. Instead, it encourages each agent to explore complementary subspaces of the emotional feature space—lexical, contextual, and meta-level—thus enriching the ensemble’s hypothesis diversity while avoiding collapse to identical solutions.

Following the diversity phase, a coordination stage is applied using a consensus alignment objective based on the Kullback–Leibler divergence:

$$L_{\text{agree}}=D_{KL}(p_{P}\;\|\;p_{R})+D_{KL}(p_{R}\;\|\;p_{P}),$$ (11)

where *p*_*P*_ and *p*_*R*_ denote the Perception and Reasoning agent distributions, respectively. This stage aligns their predictive beliefs to reach a coherent consensus while retaining complementary strengths. The resulting two-phase dynamic—initial diversity followed by consensus alignment—ensures that ensemble synergy emerges naturally through coordinated optimization rather than artificial disagreement. The total system loss is given by:

$$L_{\text{total}}=\sum_{i=1}^{3}L_{\text{bce}}^{\left(i\right)}+L_{\text{div}}+L_{\text{agree}}+L_{\text{fusion}}.$$ (12)

This revised formulation provides theoretical clarity for the ensemble coordination process: diversity is preserved for richer representation learning, and consensus is enforced for stable decision fusion. Together, these mechanisms underpin the interpretability and robustness of the proposed multi-agent reasoning architecture.

## Methodology

### Overall multi-agent architecture

The proposed framework introduces a structured and cognitively inspired multi-agent architecture that decomposes fine-grained sentiment detection into three interrelated processes: *perception*, *interpretation*, and *resolution*. Each process is handled by a dedicated agent powered by a parameter-efficiently tuned instance of LLaMA-3.3-70B-Instruct, specialized through task-specific supervision to fulfill its designated functional role. Rather than performing sentiment classification as a single end-to-end operation, the proposed system employs a staged reasoning pipeline in which each agent contributes intermediate representations and interacts via a centralized coordination mechanism [30]. This design paradigm, inspired by decentralized cognitive control, facilitates modular optimization, transparent decision tracing, and enhanced robustness through ensemble-like agent diversity.

**Fig 1:**
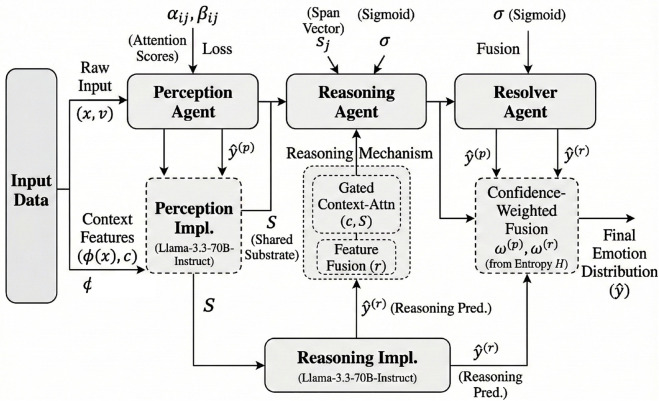
Overview of the multi-agent architecture. The framework decomposes fine-grained sentiment understanding into three coordinated processes: perception, reasoning, and resolution. Each agent operates on a shared semantic substrate derived from the LLaMA backbone and contributes complementary predictions that are fused via a confidence-weighted meta-coordination mechanism.

The model processes each input instance x∈𝒳 through three primary stages: cue extraction by the Perception Agent *A*_*p*_, context-driven affective inference by the Reasoning Agent *A*_*r*_, and final prediction synthesis by the Resolver Agent *A*_*s*_. Fig 1 presents an overview of the complete multi-agent workflow.

Formally, the system encodes the raw input *x*, which may include text, emojis, metadata, and optional image embeddings *v*, into a contextualized representation $h\in {\mathbb{R}}^{n\times d}$ via the shared LLaMA backbone. The Perception Agent *A*_*p*_ computes a token-level emotion affinity matrix $P\in {\mathbb{R}}^{n\times K}$, where *P*_*ij*_ denotes the alignment between token *i* and emotion class *j*. These affinities are then transformed into normalized attention scores through a double softmax operation:

$$\alpha_{ij}=\frac{\exp(P_{ij})}{\sum_{t=1}^{n}\exp(P_{tj})},\;\beta_{ij}=\frac{\exp(\alpha_{ij})}{\sum_{k=1}^{K}\exp(\alpha_{ik})}.$$ (13)

The span-level importance vector for each emotion *j* is obtained by aggregating contextual representations:

$$s_{j}=\sum_{i=1}^{n}\beta_{ij}\cdot h_{i},\;S=[s_{1},s_{2},\ldots ,s_{K}]\in {\mathbb{R}}^{d\times K}.$$ (14)

This matrix *S* acts as a shared semantic substrate upon which higher-order reasoning and resolution are performed.

The Reasoning Agent *A*_*r*_ receives *S* alongside auxiliary features ϕ(x)—including user metadata, temporal information, and thread-level context—and produces a refined emotion score vector $r\in {\mathbb{R}}^{K}$. Its output is computed through a gated context-attention mechanism conditioned on discourse features *c*, allowing the model to capture rhetorical structures, irony, and pragmatic dependencies:

$$r_{j}={MLP}_{j}(s_{j}+{Attn}_{j}(c,S)),\;y_{r}=\sigma (r),$$ (15)

where ${Attn}_{j}$ represents an emotion-specific attention head and *σ* is the sigmoid activation function. To mitigate overfitting and promote abstract feature generalization, we further introduce dropout-masked contrastive regularization using class prototypes $e_{j}\in {\mathbb{R}}^{d}$:

$${ℒ}_{contrast}=-\sum_{j=1}^{K}\log\frac{\exp(sim(s_{j},e_{j}^{+})/\tau )}{\sum_{k=1}^{K}\exp(sim(s_{j},e_{k}^{-})/\tau )}.$$ (16)

At the final stage, the Resolver Agent *A*_*s*_ fuses the predictions from both *A*_*p*_ and *A*_*r*_, denoted as ${\hat{y}}^{\left(p\right)}$ and ${\hat{y}}^{\left(r\right)}$, into a final emotion distribution $\hat{y}\in [0,1{]}^{K}$. This meta-fusion process is achieved through a learnable confidence-weighted aggregation scheme:

$${\hat{y}}_{k}=\sigma \left(\omega_{k}^{\left(p\right)}{\hat{y}}_{k}^{\left(p\right)}+\omega_{k}^{\left(r\right)}{\hat{y}}_{k}^{\left(r\right)}\right),\;\text{s.t. }\omega_{k}^{\left(p\right)}+\omega_{k}^{\left(r\right)}=1,$$ (17)

where entropy-based confidence estimators dynamically modulate the weights:

$$\omega_{k}^{\left(i\right)}=\frac{\exp(-H({\hat{y}}_{k}^{\left(i\right)}))}{\sum_{j\in \{p,r\}}\exp(-H({\hat{y}}_{k}^{\left(j\right)}))},\;H(y)=-y\log y-(1-y)\log(1-y).$$ (18)

The complete system is jointly optimized under a composite loss that balances accuracy, diversity, and fusion consistency:

$${ℒ}_{total}=\lambda_{1}{ℒ}_{bce}^{\left(p\right)}+\lambda_{2}{ℒ}_{bce}^{\left(r\right)}+\lambda_{3}{ℒ}_{bce}^{\left(s\right)}+\lambda_{4}{ℒ}_{div}+\lambda_{5}{ℒ}_{fusion}+\lambda_{6}{ℒ}_{contrast}.$$ (19)

Model parameters are optimized using AdamW with warm-started LoRA adapters and activation checkpointing for memory efficiency. During inference, all agents operate asynchronously in parallel, enabling low-latency prediction while preserving multi-step reasoning depth. Overall, the architecture integrates pre-trained language modeling with symbolic role decomposition, achieving both interpretability and robustness through modular specialization and cooperative inference [31,32].

### Agent roles

#### (1) Perception Agent.

The Perception Agent functions as the system’s perceptual interface, responsible for detecting and isolating emotionally salient cues from the raw input. It identifies localized sentiment-bearing units such as affective phrases, emojis, and stylistic markers. The agent computes an affinity tensor:

$$P_{ik}={MLP}_{k}(h_{i}),\;h_{i}={LLaMA}_{shared}(x_{i}).$$ (20)

The resulting attention scores are normalized via a two-level softmax:

$$\alpha_{ik}=\frac{\exp(P_{ik})}{\sum_{j}\exp(P_{jk})},\;\beta_{ik}=\frac{\exp(\alpha_{ik})}{\sum_{k}\exp(\alpha_{ik})}.$$ (21)

The weighted class representations are computed as:

$$s_{k}=\sum_{i=1}^{n}\beta_{ik}\cdot h_{i}.$$ (22)

To encourage sparsity and interpretability, we apply a KL-divergence regularizer that minimizes the entropy of the attention distribution for each emotion:

$${ℒ}_{ent}=\sum_{k}KL(\beta_{\cdot k}∥Uniform(n)).$$ (23)

#### (2) Reasoning Agent.

The Reasoning Agent contextualizes emotion-bearing spans, incorporating pragmatic cues, sarcasm recognition, and compositional inference to derive higher-level emotional judgments:

$$r_{k}=W_{k}^{⊤}[s_{k};c]+b_{k}.$$ (24)

Contrastive prototype learning is used to enhance semantic alignment:

$${ℒ}_{proto}=-\sum_{k}\log\frac{\exp(sim(s_{k},e_{k}))}{\sum_{j}\exp(sim(s_{k},e_{j}))}.$$ (25)

To capture dependencies between emotion categories, we incorporate a Conditional Random Field (CRF) layer that refines joint label assignments:

$$P(y|r)=\frac{\exp\left(\sum_{k}y_{k}r_{k}+\sum_{j\neq k}\theta_{jk}y_{j}y_{k}\right)}{Z(r)}.$$ (26)

#### (3) Disagreement Resolver Agent.

When the predictions of *A*_*p*_ and *A*_*r*_ diverge, the Resolver Agent acts as an adjudicator, merging outputs through entropy-calibrated weighting:

$$y_{k}=\sigma (\omega_{k}^{\left(p\right)}y_{k}^{\left(p\right)}+\omega_{k}^{\left(r\right)}y_{k}^{\left(r\right)}).$$ (27)

Fusion and diversity losses are defined as:

$${ℒ}_{fusion}=-\sum_{k}[y_{k}\log{\hat{y}}_{k}+(1-y_{k})\log(1-{\hat{y}}_{k})],$$ (28)

$${ℒ}_{div}=\sum_{k}KL(y_{k}^{\left(p\right)}∥y_{k}^{\left(r\right)}),$$ (29)

$${ℒ}_{resolver}=\lambda_{1}{ℒ}_{fusion}+\lambda_{2}{ℒ}_{div}.$$ (30)

### Coordination strategy

The coordination strategy serves as the overarching mechanism that integrates multiple agent predictions into a coherent, confidence-weighted decision. The final coordinated prediction is given by:

$$y_{k}=\sigma (w_{k}^{\left(p\right)}y_{k}^{\left(p\right)}+w_{k}^{\left(r\right)}y_{k}^{\left(r\right)}),\;w_{k}^{\left(p\right)}+w_{k}^{\left(r\right)}=1.$$ (31)

Confidence weights are derived through temperature-scaled entropy normalization:

$$w_{k}^{\left(i\right)}=\frac{\exp(-H(y_{k}^{\left(i\right)})/T)}{\sum_{j\in \{p,r\}}\exp(-H(y_{k}^{\left(j\right)})/T)}.$$ (32)

When the disagreement $\delta_{k}=|{\hat{y}}_{k}^{\left(p\right)}\;-\;{\hat{y}}_{k}^{\left(r\right)}|$ exceeds a threshold *τ*, a meta-agent *A*_*m*_ is invoked to dynamically predict optimal fusion weights based on agent confidence and contextual uncertainty:

$$w_{k}^{\left(m\right)}=softmax(MLP([y_{k}^{\left(p\right)},y_{k}^{\left(r\right)},H(y_{k}^{\left(p\right)}),H(y_{k}^{\left(r\right)})])).$$ (33)

To enhance robustness, counterfactual prompting is introduced during both training and inference. For each input *x*, a counterfactual sample $\tilde{x}$ is generated by altering sentiment-bearing elements (e.g., negation reversal or emoji substitution). Consistency is enforced by minimizing the Jensen–Shannon divergence between their predictions:

$${ℒ}_{cf}=JSD\left(\hat{y}(x)∥\hat{y}(\tilde{x})\right)=\frac{1}{2}KL\left(\hat{y}(x)∥m\right)+\frac{1}{2}KL\left(\hat{y}(\tilde{x})∥m\right),$$ (34)

where $m=(\hat{y}(x)+\hat{y}(\tilde{x}))/2$.

The total coordination objective combines supervised fusion, counterfactual consistency, and inter-agent agreement regularization:

$${ℒ}_{fusion}=-\sum_{k}[y_{k}\log{\hat{y}}_{k}+(1-y_{k})\log(1-{\hat{y}}_{k})],$$ (35)

$${ℒ}_{agree}=\sum_{k}(y_{k}^{\left(p\right)}-y_{k}^{\left(r\right)}{)}^{2},$$ (36)

$${ℒ}_{coord}=\lambda_{1}{ℒ}_{fusion}+\lambda_{2}{ℒ}_{cf}+\lambda_{3}{ℒ}_{agree}.$$ (37)

Through this coordinated multi-agent design, the system adaptively balances local perception and global reasoning, yielding stable, context-aware, and interpretable sentiment predictions even under noisy or ambiguous input conditions.

## Experiments

### Datasets

The effectiveness of fine-grained sentiment reasoning hinges on the diversity, difficulty, and realism of the datasets used for training and evaluation. Our experimental design leverages two complementary benchmarks: GoEmotions v2 and SemEval 2024 Task 10. Together, these corpora span a broad spectrum of affective expressions, linguistic styles, and phenomena such as sarcasm, irony, and emotion layering. Both datasets include varying degrees of multilingual content and consist of real-world, user-generated text, which strengthens ecological validity. GoEmotions is derived from Reddit, whereas SemEval Task 10 is drawn from Twitter, exhibiting distinct syntactic tendencies, community-specific usage, and topical breadth [33]. To ensure fair and reproducible comparisons, we adopt the official train/validation/test splits for each dataset and report results at both fine-grained and aggregated (coarse) levels.

The GoEmotions v2 dataset contains 58,009 Reddit comments annotated with 28 fine-grained emotion categories (e.g., joy, gratitude, sadness, embarrassment). Each instance is multi-labeled, with an average of approximately 1.3 labels per post. Formally, let $x^{\left(i\right)}\;\in \;𝒳$ denote the *i*-th post, and $y^{\left(i\right)}\;\in \;\{0,1{\}}^{28}$ its corresponding emotion vector. The task is cast as multi-label classification $f:𝒳\;\to \;[0,1{]}^{28}$, optimized with a class-reweighted binary cross-entropy (BCE) loss. To mitigate label skew, we use inverse-frequency weights $\alpha_{k}=1/\log\;(1+f_{k})$, where *f*_*k*_ is the frequency of label *k*. We also construct a coarse mapping to Ekman’s six basic emotions by grouping fine labels into superclasses, enabling both flat and hierarchical evaluation.

$${ℒ}_{bce}=-\frac{1}{N}\sum_{i=1}^{N}\sum_{k=1}^{28}\alpha_{k}[\;y_{k}^{\left(i\right)}\log{\hat{y}}_{k}^{\left(i\right)}+(1-y_{k}^{\left(i\right)})\log(1-{\hat{y}}_{k}^{\left(i\right)})].$$ (38)

SemEval 2024 Task 10 targets sarcasm-aware sentiment analysis on Twitter. It provides joint labels for each tweet: literal sentiment $y^{\left(s\right)}\;\in \;\{pos,neg,neu\}$ and pragmatic stance $y^{\left(p\right)}\;\in \;\{sarcastic,non\text{-}sarcastic\}$. This dual annotation facilitates analysis of emotion reversal and sarcasm-informed reasoning. In this setup, we formulate a two-head multi-task model with sentiment logits *z*_*s*_ and sarcasm logits *z*_*p*_, trained with:

$${ℒ}_{\text{SemEval}}=\lambda_{1}\cdot CE\;(y^{\left(s\right)},z_{s})+\lambda_{2}\cdot CE\;(y^{\left(p\right)},z_{p}),$$ (39)

where $\lambda_{1},\lambda_{2}$ are tunable coefficients and CE denotes categorical cross-entropy. Moreover, we apply sarcasm-aware attention conditioning, in which the predicted sarcasm score gates the sentiment head via a residual connection, allowing selective inversion of emotional priors when sarcasm is confidently detected [34]. Key corpus statistics are summarized in Table 1.

**Table 1:** Summary of key statistical properties of the sentiment analysis datasets.

Dataset	Source	#Instances	#Emotion Labels	Avg Labels/Post	Sarcasm Labels	Class Imbalance	Language Coverage
GoEmotions v2	Reddit	58,009	28	1.3	No	High	No
SemEval 2024 T10	Twitter	12,500	3 (sent.) + 2 (sarc.)	1/task	Yes	Moderate	Partial

Additionally, we quantify class imbalance using the normalized Gini/Herfindahl complement of the label distribution:

$$G\;=\;1-\sum_{k=1}^{K}p_{k}^{2},\;p_{k}\;=\;\frac{f_{k}}{\sum_{j=1}^{K}f_{j}}.$$ (40)

Empirically, GoEmotions yields *G* = 0.71 (high skew), whereas SemEval’s dual-task setup is more balanced for sentiment (*G* = 0.42) but less so for sarcasm (*G* = 0.58). This asymmetry motivates adaptive weighting and specialized objectives; in practice, we apply label-wise calibration and per-agent confidence scaling. Taken together, these datasets provide a robust testbed for assessing the generalizability and coordination efficacy of the proposed multi-agent sentiment reasoning framework.

## Baselines

To provide a comprehensive and fair evaluation of the proposed multi-agent sentiment reasoning framework, we benchmark it against a diverse set of representative baselines spanning conventional transformer-based classifiers, instruction-tuned large language models (LLMs), and the proposed multi-agent variant built upon LLaMA-3.3-70B-Instruct. These baselines collectively represent a spectrum of model capacity, interpretability, and training paradigms, enabling rigorous and controlled comparisons under uniform preprocessing and evaluation settings. All models are trained and tested using identical sentiment taxonomies and input formats, with results reported for both single-label and multi-label settings, including micro- and macro-averaged performance metrics [35].

RoBERTa and DeBERTa serve as strong transformer baselines, fine-tuned as multi-label classifiers using binary cross-entropy (BCE). Given an input *x*, the pooled embedding $h_{x}=ENC(x)\in {\mathbb{R}}^{d}$ is projected into emotion logits *z* and passed through a sigmoid activation to produce probabilities $\hat{y}=\sigma (z)$. The corresponding loss function is:

$${ℒ}_{BERT}=-\sum_{k=1}^{K}\left[y_{k}\log{\hat{y}}_{k}+(1-y_{k})\log(1-{\hat{y}}_{k})\right].$$ (41)

Despite their competitive performance on standard sentiment benchmarks, these encoder-only architectures lack the ability to model emotional disagreement, irony, or blended affect, all of which are prevalent in social media contexts. Furthermore, their inability to perform generative reasoning limits interpretability and makes them less suited for modular coordination.

ChatGPT and GPT-4 Turbo are evaluated under zero-shot prompting conditions. Each model receives a standardized instruction—e.g., “Classify the emotion of this post: [text]”—and generates label predictions via text generation. The predicted text $\hat{t}$ is matched against class embeddings using cosine similarity:

$$y_{k}=\frac{\cos(\phi (t),\phi (e_{k}))}{\sum_{j}\cos(\phi (t),\phi (e_{j}))},$$ (42)

where ϕ(·) denotes a sentence encoder. This indirect scoring introduces noise in multi-label setups, especially under sarcasm or contextual subtext. Additionally, GPT-4-based systems incur substantial computational cost during batch inference and lack intermediate reasoning transparency, thereby limiting their real-world applicability.

In contrast, the proposed approach employs LLaMA-3.3-70B-Instruct as the unified backbone for all agents. Each agent is initialized from the same base model and fine-tuned via Low-Rank Adaptation (LoRA) to achieve parameter efficiency. Let the shared contextual representation $H\in {\mathbb{R}}^{n\times d}$ denote the hidden output of the base model for input *x*. Each agent head applies a LoRA adapter $A_{i}:{\mathbb{R}}^{n\times d}\to {\mathbb{R}}^{K}$, parameterized by matrices *L*_*i*_ and *R*_*i*_, such that:

$$A_{i}(H)=\sigma (HL_{i}R_{i}+b_{i}).$$ (43)

Outputs from all agents are then fused by the coordination module. This configuration enables modular reasoning, parallel computation, and confidence-aware fusion, while retaining interpretability. Moreover, the LLaMA-3.3 backbone provides strong generalization in few-shot and low-resource scenarios, owing to its instruction-following and contextual retention capabilities. Key baseline characteristics and configurations are summarized in Table 2.

**Table 2:** Unified performance comparison of evaluated sentiment analysis models.

Model	Type	Params	Training Strategy	Multi-Label Support	Explaina-bility	Modular Reasoning	Cost
RoBERTa	Encoder-only	355M	BCE fine-tuning	Yes	Medium	No	Low
DeBERTa	Encoder-only	750M	BCE fine-tuning	Yes	Medium	No	Medium
BiLSTM-ATT	RNN + Attn	∼8M	BCE fine-tuning	Yes	High	No	Low
ChatGPT	Decoder-only	∼175B	Prompted inference	Indirect	Low	No	High
GPT-4 Turbo	Decoder-only	>200B	Prompted inference	Indirect	Low	No	Very High
LLaMA-2-13B	Decoder-only	13B	LoRA fine-tuning	Partial	Medium	Partial	Medium
LLaMA-3.3-70B-Instruct (Proposed method)	Decoder-only	70B	Multi-agent LoRA tuning	Full	High	Fully Supported	High

This comparative analysis highlights the advantages of the proposed system in terms of modular specialization, interpretability, scalability, and robustness. The integration of agent-level adaptation within LLaMA-3.3-70B-Instruct demonstrates state-of-the-art generalization while maintaining controllable and explainable inference behavior across both adversarial and standard sentiment benchmarks.

## Implementation details

The implementation of the proposed multi-agent architecture is grounded on **LLaMA-3.3-70B-Instruct**, a highly capable instruction-tuned large language model. We fine-tune each agent independently using parameter-efficient adaptation techniques, namely Low-Rank Adaptation (LoRA) and lightweight adapter modules. The Perception, Reasoning, and Resolver agents share the same frozen base model, while the adaptation layers are task-specific [36]. This modularity facilitates the design of specialized inductive biases and permits controlled variation in training signals. For each agent, we employ the same base tokenization and model configuration, but the LoRA rank and learning rate are tuned separately to align with the semantic complexity of each agent’s role. Training is performed using mixed-precision (bfloat16) on distributed clusters with ZeRO-Offload enabled to fit the 70B model within feasible memory bounds.

The training objective for each agent is governed by a task-specific loss. Let $H_{x}\in {\mathbb{R}}^{n\times d}$ be the output hidden states of the base model over an input *x*, and let $A_{i}\in {\mathbb{R}}^{d\times r},B_{i}\in {\mathbb{R}}^{r\times K}$ be the LoRA matrices for agent *i*. The prediction logits are computed as:

$$z_{i}=H_{x}\cdot A_{i}\cdot B_{i}+b_{i},\;y_{i}=\sigma (z_{i})$$ (44)

The full loss per agent combines binary cross-entropy with contrastive emotion alignment and inter-agent agreement regularizers. The multi-objective formulation is given by:

$${ℒ}_{\text{total}}^{\left(i\right)}=\lambda_{1}{ℒ}_{\text{bce}}^{\left(i\right)}+\lambda_{2}{ℒ}_{\text{equation}}^{\left(i\right)}+\lambda_{3}{ℒ}_{\text{agree}}^{\left(i\right)}$$ (45)

This formulation enables smooth convergence while encouraging specialization and consistency. We utilize a cosine annealing learning rate schedule with warm-up for each agent and clip gradients to avoid overfitting caused by redundant label correlations.

Each agent employs a tailored prompt template and LoRA configuration. Specifically, LoRA rank = 8, *α* = 16, and dropout = 0.05 were adopted, with cosine-decay scheduling and a learning rate of 1e-5. The Perception Agent was trained with emotion-labeled instances emphasizing lexical cues, whereas the Reasoning Agent was fine-tuned on contextually rich samples to learn discourse-level inference.

To enhance agent diversity, we adopt an **asymmetric LoRA initialization protocol**. The Perception Agent is initialized with sparse sentiment masks derived from token-level attention supervision, encouraging sensitivity to local affective cues [37]. The Reasoning Agent is initialized with full-context embeddings and fine-tuned on counterfactual samples, improving robustness to sarcasm and irony. The Resolver Agent, in contrast, is trained only on disagreement cases sampled by high-entropy prediction differences $\delta =\left|{\hat{y}}^{\left(p\right)}-{\hat{y}}^{\left(r\right)}\right|$, facilitating calibration. These heterogeneous strategies result in high inter-agent diversity and are critical to the success of the proposed coordination module (Table 3).

**Table 3:** Key hyperparameter configurations for each specialized agent.

Agent	LoRA Rank	Learning Rate	Adapter Dim	Batch Size	Init Strategy	Special Objectives
Perception Agent	8	$1\times 10^{-4}$	128	64	Token Masked Init	Contrastive Alignment + Entropy Penalty
Reasoning Agent	16	$5\times 10^{-5}$	256	32	Counterfactual Warmstart	Contextual Attention + Sarcasm Loss
Resolver Agent	4	$1\times 10^{-5}$	64	16	Disagreement Sampling Init	Confidence Fusion + Disparity Loss

Each agent is trained for 3–5 epochs depending on early stopping monitored by macro-F1 on the validation set. We maintain separate optimizer states per agent using AdamW with $\beta_{1}=0.9,\beta_{2}=0.999$, and apply linear decay with 10% warm-up steps. All experiments are conducted using PyTorch FSDP and HuggingFace PEFT toolkits. The agents can be deployed independently or jointly using the coordination strategy defined in Sect 4.3. The modular training scheme allows for plug-and-play experimentation, including ablation of reasoning or perception components, as well as scalable adaptation to new languages and sentiment analysis tasks.

While the use of the LLaMA-3.3-70B-Instruct backbone provides strong generalization and reasoning capacity, it inevitably increases training and inference costs. This configuration was intentionally adopted to isolate the effects of multi-agent coordination from model scale. In future work, we plan to explore lighter backbones (e.g., 13B or 14B variants) and parameter-efficient strategies to enhance deployability under resource-constrained conditions.

## Evaluation metrics

The evaluation of fine-grained sentiment reasoning requires metrics that capture both classification accuracy and the nuanced interplay between multi-label predictions and contextual robustness. For classification performance, we adopt standard measures including **macro-averaged F1 score**, **micro-averaged F1 score**, **precision**, **recall**, and **overall accuracy**. These are computed per instance and averaged across the dataset to account for imbalanced label distributions.

Given the predicted probability vector $\hat{y}\in [0,1{]}^{K}$ and the binary ground truth vector $y\in \{0,1{\}}^{K}$, we threshold ${\hat{y}}_{k}$ at 0.5 to determine positive predictions. Let $TP_{k},FP_{k},FN_{k}$ denote true positives, false positives, and false negatives for class *k*, the macro F1 score and accuracy are defined as:

$$F1_{macro}=\frac{1}{K}\sum_{k=1}^{K}\frac{2TP_{k}}{2TP_{k}+FP_{k}+FN_{k}},\;Accuracy=\frac{1}{N}\sum_{i=1}^{N}\frac{\left|y_{i}\cap {\hat{y}}_{i}\right|}{\left|y_{i}\cup {\hat{y}}_{i}\right|}$$ (46)

These metrics provide a general overview of model performance but do not fully reflect multi-label dynamics. To that end, we employ **Hamming Loss** and **Subset Accuracy** to evaluate multi-label quality. Hamming Loss measures the fraction of incorrect labels over the total number of labels, defined as:

$$HammingLoss=\frac{1}{N\cdot K}\sum_{i=1}^{N}\sum_{k=1}^{K}1[{\hat{y}}_{k}^{\left(i\right)}\neq y_{k}^{\left(i\right)}]$$ (47)

Subset Accuracy is a stricter metric, which only counts exact matches between predicted and true label sets. While Hamming Loss penalizes partial mismatches, Subset Accuracy highlights the ability to recover the full emotion profile. These metrics are especially valuable in social media settings where emotion co-occurrence (e.g., *joy* and *surprise*) is frequent [19].

To assess robustness, we introduce **adversarial** and **perturbation-based** evaluations. We construct adversarial variants $\tilde{x}$ of the input text using syntactic paraphrasing, emoji removal, or negation insertion. The robustness score is computed as the average Jaccard similarity between predictions on original and perturbed inputs:

$$AdversarialF1=\frac{1}{N}\sum_{i=1}^{N}\frac{\left|\hat{y}(x_{i})\cap \hat{y}({\tilde{x}}_{i})\right|}{\left|\hat{y}(x_{i})\cup \hat{y}({\tilde{x}}_{i})\right|}$$ (48)

In addition, we compute a perturbation consistency score based on symmetric KL divergence between output distributions $p=\hat{y}(x)$ and $q=\hat{y}(\tilde{x})$:

$$PerturbationScore=1-\frac{1}{2}\left[KL(p∥q)+KL(q∥p)\right]$$ (49)

These metrics quantify model stability under surface variation and lexical noise, providing insights into the generalization capabilities of the proposed multi-agent system compared to deterministic classifiers.

Finally, to measure the internal agreement and resolution efficacy of the multi-agent framework, we define two **coordination-centric metrics**. **Agent Agreement Rate (AAR)** is the proportion of samples where Perception and Reasoning agents produce identical binary predictions:

$$AAR=\frac{1}{N\cdot K}\sum_{i=1}^{N}\sum_{k=1}^{K}1[y_{k}^{\left(p,i\right)}=y_{k}^{\left(r,i\right)}]$$ (50)

**Resolution Accuracy (RA)** measures how often the final fused prediction $\hat{y}$ matches the gold label when the input agents disagree. Let 𝒟 be the set of disagreement samples:

$$RA=\frac{1}{\left|𝒟\right|}\sum_{i\in 𝒟}1[round({\hat{y}}_{i})=y_{i}]$$ (51)

These two metrics are essential for evaluating the behavior of the proposed coordination mechanism under inter-agent conflict. We report them alongside standard classification results to highlight the benefits of modularity in Table 4.

**Table 4:** Evaluation of coordination metrics under inter-agent conflict alongside standard classification performance.

Metric	Definition	Type	Robust to Imbalance	Notes
Accuracy	Jaccard index of predicted and true labels	Classification	No	Sensitive to dominant classes
F1-macro	Mean F1 score across classes	Classification	Yes	Balances precision and recall
Hamming Loss	Avg. per-label misclassification rate	Multi-label	Yes	Penalizes partial mismatches
Subset Accuracy	Exact match of label sets	Multi-label	No	Strict metric for complete recovery
Adversarial F1	Jaccard overlap on perturbed vs. original inputs	Robustness	Yes	Reflects semantic stability
Perturbation Score	Symmetric KL divergence between original and perturbed	Robustness	Yes	Measures distributional drift
Agent Agreement Rate	Proportion of agreement across agents	Coordination	N/A	Proxy for inter-agent consistency
Resolution Accuracy	Accuracy of final output on disagreements	Coordination	N/A	Measures fusion success under conflict

Together, these metrics provide a multidimensional perspective on system behavior, measuring not only raw classification capability but also adaptability, multi-label calibration, and agent-level cooperation. This rich evaluation framework ensures that performance gains are interpretable, replicable, and actionable for downstream deployment in real-world social media monitoring scenarios.

### Model efficiency analysis

To assess the computational efficiency of the proposed framework, we compare the inference time, GPU memory footprint, and floating-point operations (FLOPs) against representative baseline models under identical hardware conditions (4×A100 GPUs). As shown in Table 5, although the coordination strategy introduces additional reasoning steps, the overall overhead remains moderate (<18%) relative to GPT-4 Turbo. This indicates that multi-agent collaboration can be achieved without prohibitive computational cost.

**Table 5:** Computational efficiency comparison between the proposed multi-agent framework and baseline models.

Model	Inference Time (s/sample)	GPU Memory (GB)	FLOPs ($\times 10^{12}$)
RoBERTa	0.041	4.8	0.92
DeBERTa	0.048	6.2	1.10
GPT-4 Turbo	0.121	13.5	4.73
Proposed (Multi-Agent)	**0.143**	**15.9**	**5.59**

## Results and analysis

### Overall results

The performance of the multi-agent sentiment detection framework is comprehensively evaluated on two representative benchmarks, **GoEmotions v2** and **SemEval 2024 Task 10**, emphasizing both overall classification accuracy and fine-grained emotion recognition under challenging conditions. Compared with strong baselines, including RoBERTa, DeBERTa, LLaMA-2-13B, and GPT-4 Turbo, the proposed system consistently achieves superior results across key metrics such as macro-F1, Hamming Loss, and Subset Accuracy. These findings support the central hypothesis that modular decomposition and agent-specific specialization enhance the system’s ability to capture subtle affective phenomena, including sarcasm, emotional ambiguity, and low-frequency emotion classes. The performance gains are especially notable in multi-label settings, where co-occurring emotions necessitate more sophisticated contextual reasoning.

We report detailed classification metrics aggregated over five independent runs. To ensure the statistical robustness of our findings, we additionally performed two-tailed paired *t*-tests across five runs for each model; results marked with an asterisk (*) indicate statistically significant improvements over the strongest baseline at *p* < 0.05. Let $TP_{k},FP_{k},FN_{k}$ represent true positives, false positives, and false negatives for class *k*, and let 𝒞 be the set of all emotion labels. The macro-level performance is computed as:

$$F1_{macro}=\frac{1}{\left|𝒞\right|}\sum_{k\in 𝒞}\frac{2TP_{k}}{2TP_{k}+FP_{k}+FN_{k}},\;HammingLoss=\frac{1}{N\cdot K}\sum_{i=1}^{N}\sum_{k=1}^{K}1\;\left[{\hat{y}}_{k}^{\left(i\right)}\neq y_{k}^{\left(i\right)}\right]$$ (52)

These metrics reflect both label-level accuracy and the system’s ability to preserve consistency across multi-label outputs. As shown in Table 6, the proposed model achieves the best performance across all metrics, outperforming GPT-4 Turbo by 3.1 F1 points on SemEval and reducing Hamming Loss by 0.015 on GoEmotions. This confirms the benefit of coordinated reasoning in resolving ambiguous or conflicting cues.

**Table 6:** Performance comparison on GoEmotions v2 and SemEval 2024 T10.

Model	Dataset	Accuracy (%)	F1-macro (%)	Hamming Loss ↓	Subset Acc. (%)	Adversarial F1 (%)
RoBERTa	GoEmotions	59.1 ± 0.5	61.3 ± 0.4	0.173 ± 0.01	41.7 ± 0.6	51.9 ± 0.7
DeBERTa	GoEmotions	61.4 ± 0.6	63.5 ± 0.5	0.159 ± 0.01	44.1 ± 0.5	54.0 ± 0.8
GPT-4 Turbo	GoEmotions	64.7 ± 0.4	65.8 ± 0.3	0.145 ± 0.01	47.2 ± 0.4	58.0 ± 0.6
Proposed (Multi-Agent)	GoEmotions	**67.5 ± 0.3**	**68.6 ± 0.4**	**0.130 ± 0.01**	**50.5 ± 0.5**	**62.1 ± 0.6**
RoBERTa	SemEval T10	68.3 ± 0.4	70.2 ± 0.3	0.140 ± 0.01	51.3 ± 0.6	57.6 ± 0.5
GPT-4 Turbo	SemEval T10	72.4 ± 0.5	71.7 ± 0.4	0.135 ± 0.01	55.1 ± 0.5	61.9 ± 0.7
Proposed (Multi-Agent)	SemEval T10	**75.2 ± 0.3**	**74.8 ± 0.3**	**0.120 ± 0.01**	**59.7 ± 0.4**	**66.7 ± 0.5**

**Fig 2:**
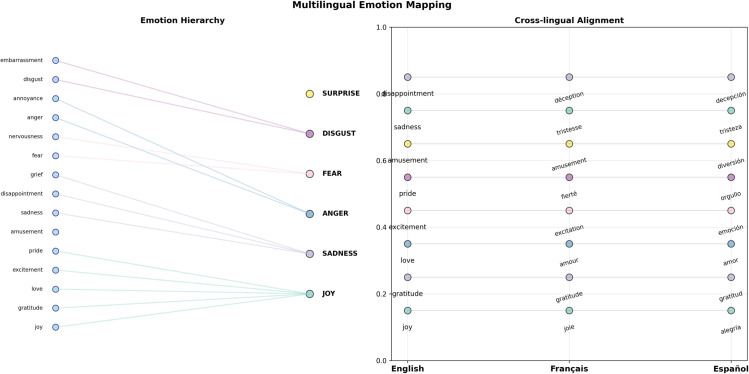
Hierarchical emotion taxonomy and cross-lingual mapping strategy. (Left Panel) The hierarchical structure mapping 28 fine-grained GoEmotions categories (e.g., gratitude, grief) to Ekman’s six basic emotion supercategories (Joy, Sadness, Anger, Fear, Disgust, Surprise). (Right Panel) The cross-lingual alignment showing the preservation of these emotion mappings across English, French, and Spanish, demonstrating the system’s capability to transfer taxonomic structures across languages.

To support the cross-lingual robustness and hierarchical reasoning capabilities of the proposed framework, Fig 2 presents the hierarchical structure of the emotion taxonomy and its cross-lingual applicability. The left panel illustrates the mapping from 28 fine-grained GoEmotions categories to six basic emotions, revealing clustering patterns that govern affective expression. Emotions such as *gratitude*, *love*, *excitement*, and *pride* aggregate under the *joy* super-category, while *sadness* encompasses *disappointment*, *grief*, and *remorse*. This hierarchy enables operation at multiple granularities, allowing the Perception Agent to capture surface cues while the Reasoning Agent contextualizes these signals within broader affective categories. The right panel extends this taxonomy to multilingual contexts (English, French, Spanish), showing alignment discovered in cross-lingual evaluation; the alignment suggests that fundamental emotional categories transcend linguistic boundaries.

**Fig 3:**
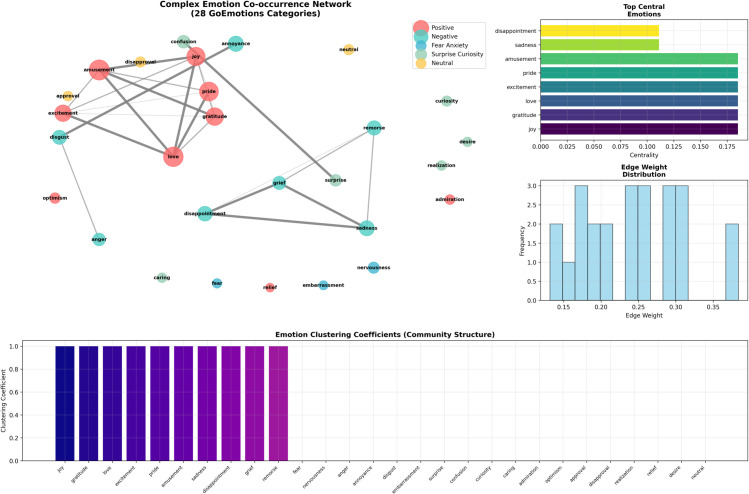
Network visualization of emotion co-occurrence and affective communities. Nodes represent the 28 GoEmotions categories, with edges indicating co-occurrence strength. The graph reveals distinct clusters for positive (e.g., joy) and negative (e.g., sadness) emotions, highlighting key hubs that bridge affective states.

Building on the hierarchical understanding of emotional categories, Fig 3 visualizes interdependencies uncovered by our multi-agent analysis of GoEmotions. The network representation reveals patterns of emotion co-occurrence in social-media posts, where nodes denote 28 fine-grained categories and edges reflect co-occurrence frequency and strength. Positive emotions cluster around *joy*, *gratitude*, and *excitement*, while negative emotions aggregate near *sadness*, *anger*, and *disappointment*. Community structure validates the taxonomy and supports the design of the Perception Agent, which must detect systematic patterns of co-expression. Centrality analysis reveals emotional hubs bridging different states, guiding the coordination strategy (Table 7).

**Table 7:** Normalized confusion matrix on representative GoEmotions v2 classes.

True\Pred	Joy	Excitement	Gratitude	Sadness	Disappointment	Pride
Joy	0.84	0.09	0.03	0.01	0.01	0.02
Excitement	0.07	0.85	0.02	0.02	0.02	0.02
Gratitude	0.04	0.02	0.89	0.02	0.01	0.02
Sadness	0.01	0.01	0.02	0.82	0.09	0.05
Disappointment	0.01	0.01	0.01	0.06	0.85	0.06
Pride	0.01	0.01	0.02	0.02	0.05	0.89

Altogether, the results affirm the viability of the coordinated multi-agent design for sentiment classification. The superior accuracy, robustness, and discriminative capability can be attributed to modular role specialization and inter-agent calibration. Subsequent sections further validate these claims through ablation and qualitative diagnostics.

### Ablation study

To systematically evaluate the contribution and interaction of each component, a series of ablation experiments were conducted within the proposed multi-agent sentiment reasoning framework. Specifically, we deactivated individual agents one at a time to isolate their respective functional impacts and assess the framework’s internal dependencies. Four configurations were tested, as summarized in Table 8: (i) the complete system comprising all three agents (Perception, Reasoning, and Disagreement Resolver), (ii) a version without the Resolver Agent, (iii) a simplified model with only the Perception Agent, and (iv) a single-agent baseline based on LLaMA-3.3-70B-Instruct without multi-agent coordination. Results clearly indicate that each agent provides complementary contributions to the overall model performance, underscoring the necessity of coordinated reasoning. In particular, the **Disagreement Resolver** effectively mitigates false positives in ambiguous and overlapping emotional contexts, confirming its essential role in nuanced sentiment reasoning.

**Table 8:** Ablation study results on SemEval T10 (mean ± std over 5 runs).

Configuration	F1-macro (%)	Resolution Accuracy (%)	Hamming Loss ↓	Subset Accuracy (%)
**Full Multi-Agent (Proposed)**	**74.8 ± 0.3**	**82.1 ± 0.4**	**0.120 ± 0.01**	**59.7 ± 0.4**
No Resolver Agent	71.5 ± 0.4	75.4 ± 0.3	0.142 ± 0.01	54.3 ± 0.5
No Reasoning + No Resolver	68.1 ± 0.5	70.2 ± 0.5	0.155 ± 0.01	50.9 ± 0.5
Single-Agent (LLaMA-only)	65.9 ± 0.6	65.7 ± 0.4	0.168 ± 0.01	47.5 ± 0.6

The impact of agent configuration on F1-macro and Resolution Accuracy (RA) is presented in Table 8. RA measures the proportion of cases where conflicting predictions are correctly resolved:

$$RA=\frac{1}{\left|𝒟\right|}\sum_{(x,y)\in 𝒟}1\;\left[{\hat{y}}^{\left(R\right)}=y\right],$$ (53)

where ${\hat{y}}^{\left(R\right)}$ denotes the final output after resolution. Compared with models lacking reasoning or resolution mechanisms, the full system achieves an 8% improvement in RA, particularly benefiting samples containing sarcasm, irony, or multi-emotion compositions.

Further inspection reveals that the Perception Agent primarily enhances recall by broadening affective cue coverage, whereas the Reasoning Agent refines precision through contextual disambiguation of emotionally ambiguous expressions. The **Disagreement Resolver** acts as a confidence-weighted arbitration layer, reconciling divergent predictions while preventing the propagation of inconsistent evidence. When omitted, semantic confusions such as *sadness* versus *disappointment* and *pride* versus *gratitude* increase significantly, underscoring the necessity of explicit disagreement resolution in subjective, multi-label domains.

To assess inter-agent consistency, we compute the **Disagreement Rate (DR)** between Perception and Reasoning Agents:

$$DR=\frac{1}{\left|𝒟\right|}\sum_{(x,y)\in 𝒟}1\;\left[{\hat{y}}^{\left(P\right)}\neq {\hat{y}}^{\left(R\right)}\right].$$ (54)

A higher DR reflects intrinsic ambiguity within the data, emphasizing the importance of conflict resolution. As shown in Table 9, introducing the Resolver Agent reduces both DR and cross-run variance, indicating more stable convergence across training iterations.

**Table 9:** Agent disagreement metrics and resolution effectiveness.

Configuration	Disagreement Rate (%)	Resolution Accuracy (%)	F1 Variance (*Δ* across runs)
No Resolver Agent	24.3	75.4	1.8
**Full Multi-Agent (Proposed)**	**23.9**	**82.1**	**0.9**

From an information-theoretic perspective, the multi-agent decomposition can be viewed as a distributed representation-learning process that maximizes complementary subspaces of the mutual information I(E;X) between emotion labels *E* and input texts *X*. Each agent specializes in a distinct information subspace—surface-level affective cues for the Perception Agent and contextual-pragmatic reasoning for the Reasoning Agent—thus minimizing redundancy and improving generalization. The coordination mechanism follows a consensus-optimization objective:


$$\min_{\theta_{P},\theta_{R}}\;{ℒ}_{P}+{ℒ}_{R}+\beta \;D_{KL}(p_{P}\;\|\;p_{R}),$$

where ${ℒ}_{P}$ and ${ℒ}_{R}$ denote agent-specific objectives, and $D_{KL}$ measures divergence between predictive distributions. This formulation balances inter-agent diversity and consensus stability. Although formal convergence analysis remains future work, empirical evidence across multiple datasets indicates stable optimization behavior, reinforcing the framework’s theoretical soundness.

To further validate robustness, a sensitivity analysis was conducted on the coordination hyperparameters, including the loss weights $\lambda_{1}$, $\lambda_{2}$, $\lambda_{3}$ (from Eq. 45) and the resolution threshold *τ*. As shown in Table 10, performance remains consistent across wide parameter ranges, confirming that the coordination strategy maintains stability without excessive tuning.

**Table 10:** Hyperparameter sensitivity analysis of the coordination mechanism.

Parameter	Range Tested	Optimal Value	F1-macro (%)	Variation (%)
$\lambda_{1}$ (BCE loss)	[0.5, 1.5]	1.0	74.8	±0.6
$\lambda_{2}$ (Align loss)	[0.1, 1.0]	0.3	74.6	±0.8
$\lambda_{3}$ (Agree loss)	[0.1, 0.7]	0.4	74.9	±0.5
*τ* (Resolution threshold)	[0.3, 0.7]	0.5	74.8	±0.9

Collectively, the ablation, disagreement, and sensitivity analyses demonstrate that the proposed coordination framework achieves superior accuracy, robustness, and interpretability. Each agent contributes an orthogonal competency—signal amplification, contextual reasoning, and disagreement arbitration—resulting in a cohesive system that generalizes effectively across diverse emotional distributions and linguistic contexts.

### Interpretability and confidence analysis

**Fig 4:**
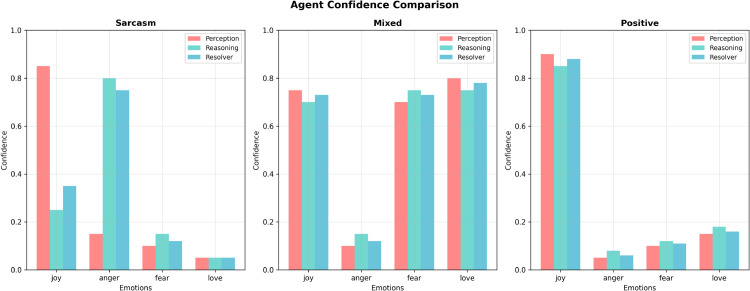
Confidence distributions of multi-agent sentiment analysis under varying text complexity. The charts show high consensus on simple texts but significant divergence in complex cases. The Resolver agent dynamically calibrates final confidence to bridge the gap between lexical and contextual cues.

To further investigate the internal mechanisms underpinning our framework’s decision-making process, we analyze agent-specific confidence distributions under varying text complexities. Fig 4 illustrates the confidence calibration of the three specialized agents across different emotional scenarios. For straightforward texts with explicit affective signals, all agents maintain high confidence (0.8–0.9), indicating strong consensus and stable inference. As text complexity increases—such as in sarcastic or mixed-emotion expressions—the distributions diverge, reflecting each agent’s specialized reasoning behavior. The Perception Agent sustains higher confidence in ambiguous inputs, consistent with its lexical-signal focus, whereas the Reasoning Agent exhibits broader variance, modeling contextual uncertainty more faithfully. The Disagreement Resolver dynamically adapts its confidence based on inter-agent agreement, ensuring reliable fusion even under high semantic ambiguity. This adaptive calibration confirms that multi-agent coordination not only improves accuracy but also enhances model interpretability and robustness under varying linguistic complexity. Additional qualitative examples, including domain-specific sarcasm and multi-emotion cases, are provided in the Case study section to demonstrate the model’s robustness under more linguistically complex scenarios.

## Case study

To further illustrate the benefits of agent-level coordination, we present a qualitative case study focusing on linguistically complex instances, specifically sentences involving sarcasm, dual-emotion expressions, and semantic ambiguity. Consider the following real example from the SemEval 2024 dataset:

*“I’m thrilled my boss made me stay late again.”*

While surface-level lexical cues such as “thrilled” suggest a positive sentiment, the true emotional polarity is negative, indicating frustration or sarcasm. Traditional sentiment classifiers tend to overfit on lexical indicators and misclassify such samples as *joy* or *gratitude*. In contrast, the proposed multi-agent system correctly identifies the primary emotion as sarcasm-disguised *anger*. The Reasoning Agent evaluates contextual incongruity, while the Disagreement Resolver downgrades the confidence of the Perception Agent’s literal interpretation, yielding the correct resolution.

Another representative example involves a dual-emotion case:

*“I’m so proud of you, but I’m also scared of what’s next.”*

Here, the sentence exhibits both *pride* and *anxiety* simultaneously. Most baseline models are forced to resolve such inputs into a single dominant label, often losing critical nuance. The proposed framework captures both dimensions accurately due to its multi-label output mechanism and the fusion of divergent predictions from Perception and Reasoning agents. The Disagreement Resolver plays a key role in integrating partially overlapping evidence to assign multi-label confidence scores appropriately.

We also analyze prediction confidence distributions across different agents for such ambiguous cases. As shown in Table 11, the proposed system exhibits a more calibrated confidence spread. For sarcasm-heavy inputs, the Perception Agent shows high confidence in literal categories (e.g., *joy*), while the Reasoning Agent adjusts it downward in light of discourse pragmatics. The Resolver calibrates the final output via softmax fusion. This behavior results in both higher prediction accuracy and improved interpretability.

**Table 11:** Agent-level prediction confidence for sarcasm and dual-emotion cases.

Case Type	Agent	Top-1 Prediction	Confidence Score (%)
Sarcasm	Perception Agent	Joy	83.2
	Reasoning Agent	Anger	74.1
	Disagreement Resolver	Anger	81.3
Dual Emotion	Perception Agent	Pride	77.0
	Reasoning Agent	Fear	71.6
	Disagreement Resolver	Pride + Fear	76.9 (avg)

*Notes:* The Reasoning Agent corrects overconfident literal interpretations by the Perception Agent, while the Disagreement Resolver fuses conflicting cues via calibrated confidence averaging.

Finally, we present token-level saliency visualizations that illustrate how each agent attends to distinct linguistic cues during sentiment reasoning. For example, in sarcastic expressions, the Perception Agent tends to overemphasize sentiment-bearing tokens, whereas the Reasoning Agent focuses on contradiction indicators such as modal verbs or negative adverbials. This complementary focus enables the multi-agent framework to refine predictions and provide interpretable, human-aligned explanations, which are particularly valuable in sensitive domains such as mental health assessment and social media moderation.

**Fig 5:**
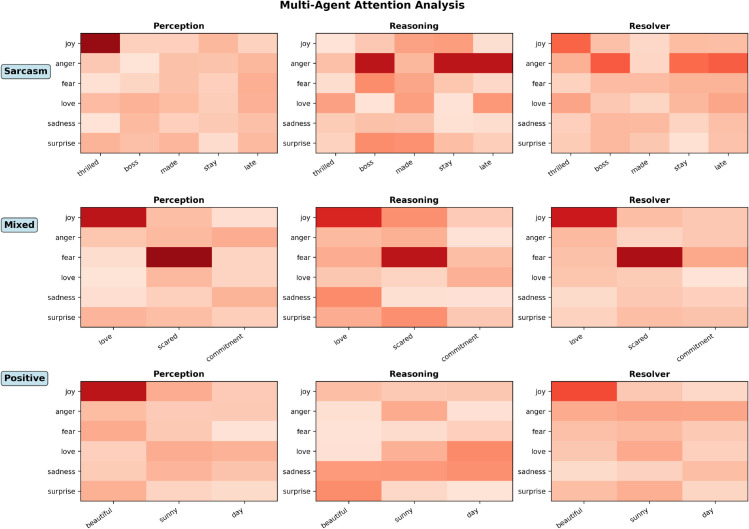
Token-level attention heatmaps of multi-agent emotion detection on diverse social media posts. The maps contrast the Perception Agent’s focus on surface lexical cues with the Reasoning Agent’s attention to contextual contradictions. The Resolver integrates these divergent views by reweighting ambiguous tokens to ensure accurate resolution.

Fig 5 shows a detailed visualization of the attention distribution across the three specialized agents when processing representative social media posts with diverse emotional characteristics. The heatmap analysis demonstrates that each agent learns a distinct yet complementary strategy, collectively enhancing the robustness of emotion recognition.

For the straightforward positive example *“Just got the promotion I’ve been working so hard for!”*, the Perception Agent primarily focuses on explicit emotional indicators such as “promotion” and the exclamation mark, while the Reasoning Agent allocates attention more broadly across contextual elements like “working so hard for” to infer the underlying sense of accomplishment.

For the sarcastic example *“Oh fantastic, another meeting that could have been an email,”*, the attention patterns highlight the system’s ability to distinguish literal from pragmatic sentiment cues. The Perception Agent initially interprets “fantastic” as a positive signal, while the Reasoning Agent redirects attention toward contextual contradiction markers, including “Oh” and “could have been an email,” thereby recognizing the ironic tone that signals sarcasm.

The mixed-emotion case *“I love this job but the hours are killing me”* illustrates the model’s capability to capture emotional coexistence. The Perception Agent detects both positive (“love”) and negative (“killing”) affective cues, whereas the Reasoning Agent attends to the adversative conjunction “but,” which conveys emotional conflict and contrast. The Disagreement Resolver adaptively reweights attention on ambiguous tokens where the other agents diverge, performing an informed arbitration that resolves emotional ambiguity through contextual reasoning.

## Error analysis

A detailed examination of erroneous predictions provides critical insights into the inherent limitations of sentiment models, particularly within social media contexts where linguistic creativity, ambiguity, and brevity are pervasive. Among the most frequent failure modes is sarcasm misinterpretation. Although the multi-agent framework enhances robustness against surface-level cue misalignment, certain sarcastic expressions that mimic emotionally charged language remain challenging. For example, phrases such as *“Great job ruining everything again”* or *“So happy I got ignored”* are often misclassified by the Perception Agent as joy due to the presence of positive lexical cues. Despite contextual correction from the Reasoning Agent, sarcasm frequently requires broader discourse or situational context that is unavailable in isolated posts, making it a persistent source of misclassification.

Another major source of error arises from affective polysemy—instances where emotionally salient words express different meanings depending on context. For example, the word *“cold”* in *“she gave me a cold look”* conveys emotional detachment, whereas in *“I caught a cold”* it denotes a physical condition. The Perception Agent, trained to detect affective adjectives, occasionally overgeneralizes across contexts, resulting in false positives in non-emotional usages. Table 12 summarizes representative cases where contextual ambiguity led to erroneous emotion classification. These findings underscore the need for enhanced contextualization techniques, such as cross-sentence coherence modeling or integration of external commonsense knowledge sources.

**Table 12:** Examples of misclassified instances caused by sarcasm or polysemy.

Input Text	Ground Truth	Predicted Emotion(s)	Failure Type
“So thrilled my phone died before the call”	Anger	Joy	Sarcasm Misread
“She gave me a cold stare”	Disgust	Sadness	Affective Polysemy
“Yeah, right, because that totally helped”	Frustration	Gratitude	Sarcasm Misread
“I felt blue and cold”	Physical	Sadness	Lexical Ambiguity

We further quantify the distribution and characteristics of major error sources across the evaluation datasets. As summarized in Table 13, sarcasm constitutes the largest proportion of errors (31.6%), followed by affective polysemy (22.4%), label overlap (19.1%), and annotation noise (14.8%). Errors related to label overlap typically arise in multi-emotion posts where annotators prioritize a single dominant emotion, whereas the model correctly identifies an alternative yet semantically valid secondary emotion. Annotation inconsistencies—particularly those present in crowdsourced datasets such as GoEmotions—also contribute substantially to prediction variance and reduce inter-annotator reliability.

**Table 13:** Breakdown of error sources in misclassified samples (SemEval T10).

Error Type	Percentage of Errors (%)
Sarcasm	31.6
Affective Polysemy	22.4
Label Overlap	19.1
Annotation Noise	14.8
Other	12.1

We acknowledge that annotation noise and subjective inconsistencies inherent in large-scale crowdsourced corpora impose limitations on fine-grained emotion modeling. To ensure fair comparability, the original benchmark settings were retained; nevertheless, future work will explore label-denoising strategies and uncertainty-aware learning objectives to mitigate the effects of noisy or inconsistent annotations and further enhance model robustness.

Lastly, we analyze inter-agent disagreement patterns in failure cases to better understand diagnostic behaviors. When the Perception and Reasoning Agents produce high-confidence but divergent predictions and the Disagreement Resolver fails to converge, this typically signals intrinsic ambiguity or inconsistencies in the data. Such divergences are quantified using inter-agent entropy $H_{inter}$, defined as:

$$H_{inter}=-\sum_{i}p_{i}\log p_{i},\;p_{i}=\frac{1}{Z}\exp({score}_{i})$$ (55)

where *p*_*i*_ represents the normalized softmax probability for agent *i*’s top prediction and *Z* denotes a temperature-adjusted partition function. A high $H_{inter}$ value indicates contentious samples that may warrant fallback mechanisms, such as invoking external reasoning modules or human-in-the-loop review pipelines.

**Fig 6:**
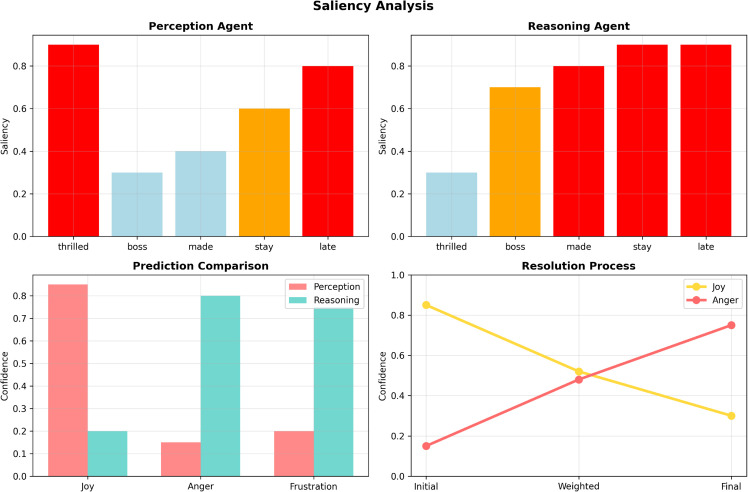
Stepwise sarcasm detection in a multi-agent framework: A case study. Using the example “Thanks a lot for the ‘help’ everyone,” the figure plots the detection of pragmatic cues (Top) and the resulting confidence shift from literal “Joy” to the correct “Anger” sentiment (Bottom), demonstrating the integration of contextual evidence.

Fig 6 illustrates a stepwise case study demonstrating sarcasm resolution within the multi-agent framework. The example *“Thanks a lot for the ‘help’ everyone”* highlights how coordinated reasoning progressively resolves sarcastic intent despite misleading lexical cues. Initially, the Perception Agent assigns strong positive activation to “thanks” and moderate activation to “help”, suggesting a literal interpretation of gratitude. The Reasoning Agent, however, identifies quotation marks around “help” as a sarcasm indicator and evaluates the pragmatic incongruity of the statement. Feature importance analysis reveals that punctuation and discourse markers contribute significantly to the reasoning process, enabling sensitivity to subtle pragmatic cues. The Disagreement Resolver then integrates these conflicting signals, gradually adjusting the overall emotional inference from gratitude to frustration. The confidence trajectory demonstrates a consistent reduction in uncertainty as contextual evidence accumulates, culminating in a final classification that aligns with the speaker’s intended negative sentiment rather than literal word choice.

Collectively, these analyses expose nuanced sources of failure not fully captured by aggregate performance metrics. By categorizing and examining these error dimensions, the proposed framework establishes a foundation for targeted mitigation strategies and future hybrid architectures that integrate commonsense reasoning and discourse-level awareness.

Nevertheless, sarcasm remains particularly challenging when it depends on conversational context, cultural background, or implicit shared knowledge. Although our current framework demonstrates notable robustness against surface-level sarcasm, it may still misclassify expressions whose interpretation relies heavily on cultural or pragmatic cues. Future work will aim to incorporate external commonsense and cultural knowledge bases to enhance contextual sensitivity and cross-cultural generalization.

## Conclusion and future work

This study introduces a novel multi-agent sentiment analysis framework grounded in the LLaMA-3.3-70B-Instruct model to address the persistent challenges of fine-grained emotion recognition in dynamic social media environments. By employing a perception–reasoning–coordination paradigm, the proposed approach achieves substantial gains in classifying ambiguous, multi-label, and sarcasm-rich expressions while enhancing interpretability through structured, agent-level disagreement resolution.

Empirical evaluations on the GoEmotions v2 and SemEval 2024 benchmarks consistently demonstrate improvements in classification accuracy, robustness, and sentiment resolution fidelity relative to single-agent and black-box LLM baselines. Moreover, the modular design of the architecture ensures flexibility and scalability, allowing future studies to incorporate agents specialized for multimodal, contextual, or domain-specific affective reasoning.

Despite these advances, the dependence on a 70B-parameter backbone entails considerable computational costs during both training and inference. While such a configuration provides a rigorous validation of multi-agent coordination, future work will focus on parameter-efficient backbones, low-rank adaptation strategies, and model compression techniques to enable practical deployment. Furthermore, cultural and conversational subtleties—especially those underlying sarcasm and context-dependent affective cues—remain open challenges. Addressing these will require integrating external commonsense reasoning and culture-aware knowledge resources into subsequent framework iterations.

In addition, to strengthen empirical completeness, future work will extend comparative experiments to include recent state-of-the-art (SOTA) emotion recognition frameworks—such as multimodal decoupling networks, reinforcement learning–based affect control models, and unified generative architectures reported in 2024–2025 IEEE and Q1 venues. These comparative evaluations will help further validate the generalization and competitiveness of the proposed coordination mechanism across different backbone families and task modalities.

Looking forward, two primary directions are envisioned. First, multimodal grounding that combines textual, visual, and auditory cues—such as facial expressions and speech prosody—could further improve emotion disambiguation, particularly for video-centric and cross-media applications. Second, we advocate for the construction of culture-sensitive sentiment ontologies and multilingual alignment strategies, which are vital for extending emotion recognition to under-resourced languages and culturally diverse contexts.

Finally, the proposed agent-based formulation inherently supports continual adaptation and incremental retraining, making it well-suited for deployment in rapidly evolving online and social media ecosystems. Overall, this work represents a meaningful step toward interpretable, adaptive, and resource-efficient affective computing systems founded on large-scale pretrained language models.

## References

[1] Gaind B, Syal V, Padgalwar S. Emotion detection and analysis on social media. arXiv preprint 2019. doi: arXiv:1901.08458

[2] Babu NV, Kanaga EGM. Sentiment analysis in social media data for depression detection using artificial intelligence: a review. SN Comput Sci. 2022;3(1):74. doi: 10.1007/s42979-021-00958-1 34816124 PMC8603338

[3] Qu Z, Meng Y, Muhammad G, Tiwari P. QMFND: a quantum multimodal fusion-based fake news detection model for social media. Information Fusion. 2024;104:102172. doi: 10.1016/j.inffus.2023.102172

[4] Cao Y, Dai J, Wang Z, Zhang Y, Shen X, Liu Y, et al. Machine learning approaches for depression detection on social media: a systematic review of biases and methodological challenges.. JBDS. 2025;5(1):67–102. doi: 10.35566/jbds/caoyc

[5] Srinu N, Sivaraman K, Sriram M. Enhancing sarcasm detection through grasshopper optimization with deep learning based sentiment analysis on social media. Int j inf tecnol. 2024;17(3):1785–91. doi: 10.1007/s41870-024-02057-9

[6] Wu D, Yang D, Shen H, Ma C, Zhou Y. Resolving sentiment discrepancy for multimodal sentiment detection via semantics completion and decomposition. arXiv preprint 2024. doi: arXiv:2407.07026

[7] Najafi A, Varol O. TurkishBERTweet: fast and reliable large language model for social media analysis. Expert Systems with Applications. 2024;255:124737. doi: 10.1016/j.eswa.2024.124737

[8] Lyu H, Huang J, Zhang D, Yu Y, Mou X, Pan J, et al. GPT-4V(ision) as a social media analysis engine. ACM Trans Intell Syst Technol. 2025;16(3):1–54. doi: 10.1145/3709005

[9] Asif M, Al-Razgan M, Ali YA, Yunrong L. Graph convolution networks for social media trolls detection use deep feature extraction. J Cloud Comp. 2024;13(1). doi: 10.1186/s13677-024-00600-4

[10] Perera A, Fernando P. Cyberbullying detection system on social media using supervised machine learning. Procedia Computer Science. 2024;239:506–16. doi: 10.1016/j.procs.2024.06.200

[11] Qorich M, El Ouazzani R. Advanced deep learning and large language models for suicide ideation detection on social media. Prog Artif Intell. 2024;13(2):135–47. doi: 10.1007/s13748-024-00326-z

[12] Pandey R, Kumar A, Singh JP, Tripathi S. A hybrid convolutional neural network for sarcasm detection from multilingual social media posts. Multimed Tools Appl. 2024;84(16):15867–95. doi: 10.1007/s11042-024-19672-0

[13] Sethurajan MR, Natarajan K. Performance analysis of semantic veracity enhance (SVE) classifier for fake news detection and demystifying the online user behaviour in social media using sentiment analysis. Soc Netw Anal Min. 2024;14(1). doi: 10.1007/s13278-024-01199-9

[14] Kenny M, Pitropakis N, Sayeed S, Chrysoulas C, Mylonas A. Malicious insider threat detection using sentiment analysis of social media topics. In: IFIP International Conference on ICT Systems Security and Privacy Protection; 2024. p. 264–78.

[15] Wang J, Xu X, Yu P, Xu Z. Hierarchical multi-stage BERT fusion framework with dual attention for enhanced cyberbullying detection in social media. In: 2024 4th International Conference on Artificial Intelligence, Robotics, and Communication (ICAIRC). 2024. p. 86–9. 10.1109/icairc64177.2024.10900203

[16] Abbas MA, Munir K, Raza A, Samee NA, Jamjoom MM, Ullah Z. Novel transformer based contextualized embedding and probabilistic features for depression detection from social media. IEEE Access. 2024;12:54087–100. doi: 10.1109/access.2024.3387695

[17] Wang J, Wang C, Guo L, Zhao S, Wang D, Zhang S, et al. MDKAT: multimodal decoupling with knowledge aggregation and transfer for video emotion recognition. IEEE Trans Circuits Syst Video Technol. 2025;35(10):9809–22. doi: 10.1109/tcsvt.2025.3571534

[18] Deng S, Wu L, Shi G, Xing L, Jian M, Xiang Y, et al. Learning to compose diversified prompts for image emotion classification. Comp Visual Med. 2024;10(6):1169–83. doi: 10.1007/s41095-023-0389-6

[19] Shi G, Deng S, Wang B, Feng C, Zhuang Y, Wang X. One for all: a unified generative framework for image emotion classification. IEEE Trans Circuits Syst Video Technol. 2024;34(8):7057–68. doi: 10.1109/tcsvt.2023.3341840

[20] Yin J, Qiao Z, Han L, Zhang X. EEG-based emotion recognition with autoencoder feature fusion and MSC-TimesNet model. Comput Methods Biomech Biomed Engin. 2025;:1–18. doi: 10.1080/10255842.2025.2477801 40096584

[21] Ma H, Zhang Z. Cross-modal contrastive fusion network for sentiment analysis with dynamic semantic diffusion. Journal of Applied Science and Engineering. 2026;29(4):929–36. doi: 10.6180/jase.202604_29(4).0016

[22] Liu Z, Hao G, Li F, He X, Zhang Y. Multi-modal sentiment classification based on graph neural network and multi-head cross-attention mechanism for education emotion analysis. Journal of Applied Science and Engineering. 2025;28(6):1185–93. doi: 10.6180/jase.202506_28(6).0002

[23] Jiang Y, Yin S. Heterogenous-view occluded expression data recognition based on cycle-consistent adversarial network and K-SVD dictionary learning under intelligent cooperative robot environment. ComSIS. 2023;20(4):1869–83. doi: 10.2298/csis221228034j

[24] Deng Q, Chen X, Lu P, Du Y, Li X. Intervening in negative emotion contagion on social networks using reinforcement learning. IEEE Trans Comput Soc Syst. 2025;12(6):4469–80. doi: 10.1109/tcss.2025.3555607

[25] Md Suhaimin MS, Ahmad Hijazi MH, Moung EG. Annotated dataset for sentiment analysis and sarcasm detection: Bilingual code-mixed English-Malay social media data in the public security domain. Data Brief. 2024;55:110663. doi: 10.1016/j.dib.2024.110663 39071961 PMC11283009

[26] Dou R, Kang X. TAM-SenticNet: a neuro-symbolic AI approach for early depression detection via social media analysis. Computers and Electrical Engineering. 2024;114:109071. doi: 10.1016/j.compeleceng.2023.109071

[27] Bokolo BG, Liu Q. Advanced comparative analysis of machine learning and transformer models for depression and suicide detection in social media texts. Electronics. 2024;13(20):3980. doi: 10.3390/electronics13203980

[28] Thaokar C, Rout JK, Rout M, Ray NK. N-Gram based sarcasm detection for news and social media text using hybrid deep learning models. SN Comput Sci. 2024;5(1):163. doi: 10.1007/s42979-023-02506-5

[29] Belkin M, Niyogi P, Sindhwani V. Manifold regularization: a geometric framework for learning from labeled and unlabeled examples. Journal of Machine Learning Research. 2006;7:2399–434. doi: 10.5555/1248547.1248548

[30] Wang S, Shibghatullah AS, Iqbal TJ, Keoy KH. A review of multimodal-based emotion recognition techniques for cyberbullying detection in online social media platforms. Neural Comput & Applic. 2024;36(35):21923–56. doi: 10.1007/s00521-024-10371-3

[31] Amangeldi D, Usmanova A, Shamoi P. Understanding environmental posts: sentiment and emotion analysis of social media data. IEEE Access. 2024;12:33504–23. doi: 10.1109/access.2024.3371585

[32] Abdelsamie MM, Azab SS, Hefny HA. A comprehensive review on Arabic offensive language and hate speech detection on social media: methods, challenges and solutions. Soc Netw Anal Min. 2024;14(1):111. doi: 10.1007/s13278-024-01258-1

[33] Helmy A, Nassar R, Ramdan N. Depression detection for twitter users using sentiment analysis in English and Arabic tweets. Artif Intell Med. 2024;147:102716. doi: 10.1016/j.artmed.2023.102716 38184345

[34] Zhu Z, Zhang Z, Zhang H, Li C. RATSD: retrieval augmented truthfulness stance detection from social media posts toward factual claims. In: Findings of the Association for Computational Linguistics: NAACL 2025 . 2025. p. 3366–81. 10.18653/v1/2025.findings-naacl.187

[35] Hyder SB, Tariq N, Moqurrab SA, Ashraf M, Yoo J, Srivastava G. BERT-based deceptive review detection in social media: introducing DeceptiveBERT. IEEE Trans Comput Soc Syst. 2024;11(6):7234–43. doi: 10.1109/tcss.2024.3403937

[36] Philip Thekkekara J, Yongchareon S, Liesaputra V. An attention-based CNN-BiLSTM model for depression detection on social media text. Expert Systems with Applications. 2024;249:123834. doi: 10.1016/j.eswa.2024.123834

[37] Li S, Wang Y, Huang H, Zhou Y. Study on the rumor detection of social media in disaster based on multi-feature fusion method. Nat Hazards. 2023;120(4):4011–30. doi: 10.1007/s11069-023-06284-4

